# MicroRNA-141-3p and microRNA-200a-3p regulate α-melanocyte stimulating hormone-stimulated melanogenesis by directly targeting microphthalmia-associated transcription factor

**DOI:** 10.1038/s41598-020-58911-w

**Published:** 2020-02-07

**Authors:** Tomohiro Itoh, Kanako Fukatani, Ayaka Nakashima, Kengo Suzuki

**Affiliations:** 1 Laboratory for Molecular Chemistry of Aquatic Materials, Department of Life Sciences, Graduate School of Bioresources, Mie University, 1577 Kurimamachiya, Tsu, Mie 514-8507 Japan; 2 Laboratory for Molecular Chemistry of Aquatic Materials, Faculty of Bioresources, Mie University, 1577 Kurimamachiya, Tsu, Mie 514-8507 Japan; 3 Euglena Co., Ltd., Central Research Centre, 75-1 Ono, Tsurumi-ku, Yokohama, Kanagawa 230-0046 Japan

**Keywords:** Gene regulation, miRNAs

## Abstract

In recent years, it has been reported that non-coding RNAs, especially microRNAs (miRNAs) and long non-coding RNAs, act as melanogenesis-regulating molecules in melanocytes. We found that the expression levels of miR-141-3p and miR-200a-3p were decreased significantly by α-melanocyte-stimulating hormone (α-MSH) stimulation in mouse melanocyte B16-4A5 cells, as demonstrated by a miRNA array. Overexpression of miR-141-3p and miR-200a-3p in B16-4A5 cells suppressed melanogenesis and tyrosinase activity. Moreover, both miR-141-3p and miR-200a-3p showed direct targeting of *microphthalmia-associated transcription factor* using a luciferase reporter assay. Furthermore, topical transfection of miR-141-3p and miR-200a-3p to three-dimensional reconstructed human skin tissue inhibited α-MSH-stimulated melanin biosynthesis. Taken together, our findings indicate that downregulation of miR-141-3p and miR-200a-3p during the α-MSH-stimulated melanogenesis process acts as an important intrinsic signal. This result is expected to lead to the development of miRNA-based whitening therapeutics.

## Introduction

Human skin comprises approximately 15% of the total body weight and performs many important functions for maintaining body homeostasis. Mainly, skin plays a role as the primary defence tissue against solar ultraviolet (UV) radiation, visible light, environmental pollution, the external microbiome, and chemicals. Skin pigmentation (melanogenesis) is one of the defence mechanisms designed to protect cellular DNA from UV radiation. Tyrosinase acts as a rate-limiting enzyme in the melanogenesis process and catalyses three steps of melanin synthesis: (i) hydroxylation of tyrosine to 3,4-dihydroxyphenylalanine (DOPA), (ii) oxidation of DOPA to dopaquinone, and (iii) oxidation of 5,6-dihydroxyindolequinone. This enzyme product is further catalysed by tyrosinase-related protein 1 (TRP-1) and dopachrome tautomerase (DCT or TRP-2) to produce melanin^1–3^. Microphthalmia-associated transcription factor (MITF) controls diverse biological processes in melanocytes, such as survival, proliferation, pigmentation, invasion, and oxygen stress, by regulating downstream gene expression^4–8^. Because tyrosinase, TRP-1, and DCT are also regulated by MITF, it is considered that controlling its expression and function are potential mechanisms for developing skin whitening materials.

Recently, it was reported that approximately 98% of the genome is actively transcribed as non-coding RNAs^9^. MicroRNAs (miRNAs) are a class of small non-coding RNAs that are 18 to 25 base-pair single-strand RNA molecules. They play pivotal roles in regulating gene expression, mainly at the post-transcriptional level. Generally, miRNAs bind imperfectly to the 3′-untranslated region (UTR) of target mRNAs and suppress subsequent protein synthesis. Many reports indicate that miRNAs are closely implicated in diverse biological processes such as cell proliferation, cell differentiation, carcinogenesis, apoptosis, diabetes, and angiogenesis^10–12^. Furthermore, miRNA expression profiles differ between cancer tissues, and changes of cancer specific miRNAs can be a diagnostic biomarker for early detection and treatment^13–15^.

In the field of dermatology, miRNAs are implicated in the aetiology of many skin diseases^16–18^. The number of hits returned for miRNA and skin in PubMed have gradually increased and is very similar to the number returned for miRNA^19^. For example, miR-31 is highly expressed in psoriatic skin keratinocytes and modulates cytokine/chemokine expression such as interleukin (IL)-1β, C-X-C motif chemokine ligand (CXCL) 1, CXCL5, and CXCL8/IL-8 via the direct targeting of serine/threonine kinase 40, which is a negative regulator of nuclear factor-kappa B^20^. MiR-23a-3p regulates the moisture content of skin through targeting hyaluronan synthase 2 in human skin samples and fibroblasts^21^. Several miRNAs (e.g. miR-let-7a, miR-7, miR-26a, miR-29, miR-206, miR-133b, miR-150, miR-196a, miR-203, miR-205, miR-382, and miR-4269) are closely associated with transforming growth factor-β signalling molecules in skin fibrosis^16,22,23^. With respect to pigmentation of the skin, numerous miRNAs regulate enzymes for melanin synthesis and melanogenesis signalling molecules^24–28^. Many of these target *MITF* and consequently regulate the mRNA levels of enzymes for melanin synthesis. Representative examples targeting *MITF* include: miR-25, miR-137, miR-145, miR-148-3p, miR-155, miR-182, miR-218, miR-508-3p, and miR-3196^29–32^. Thus, controlling the expression of *MITF* in melanocytes by miRNAs may be beneficial for treating skin pigmentation disorders such as vitiligo and melasma.

This study is the first to examine the ability of miR-141-3p and miR-200a-3p to suppress α-MSH-stimulated melanogenesis through direct inhibition of *Mitf* expression. The findings establish that modulating both miR-141-3p and miR-200a-3p may be a potential strategy for treating skin pigmentation disorders or whitening.

## Results

### Expression profiles of miR-141 and miR-200a in α-MSH-stimulated B16-4A5 murine melanoma cells

**Figure 1:**
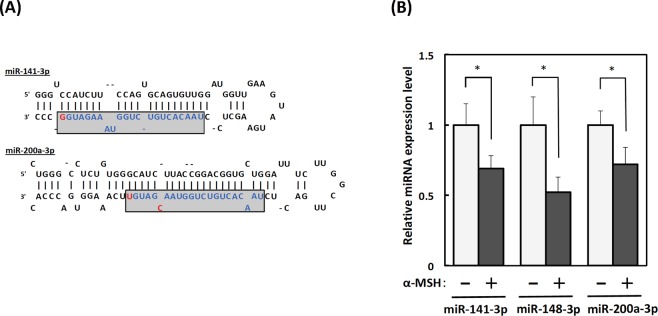
Downregulation of miR-141-3p and miR-200a-3p in α-melanocyte stimulating hormone (α-MSH)-induced B16-4A5 melanoma cells. (**A**) Sequences of miR-141-3p and miR-200a-3p. (**B**) Changes of miR-141-3p, miR-148-3p, and miR-200a-3p expression levels in α-MSH-induced B16-4A5 melanoma cells. Cells were treated with α-MSH (2 µM) for 24 h, then total RNA was extracted by TRIzol with DNase I treatment. Expression levels of miR-141-3p, miR-148-3p, and miR-200a in α-MSH-stimulated cells were examined by the TaqMan^®^miRNA assay using the real-time polymerase chain reaction. Relative ratios are shown with the value of cells without α-MSH treatment designated as 1. Data are expressed as means ± SE of three separate experiments, each performed in triplicate. Statistical comparisons were performed using the Student’s *t*-test, and differences were considered significant at p < 0.05.

To investigate the expression profiles of miRNAs in α-MSH-stimulated B16-4A5 cells, we subjected total RNA extracted from cells treated with or without α-MSH to 3D-Gene^®^ mouse miRNA Oligo chips. The expression levels of 13 miRNAs (log2 ratios >2 or <−2) were distinct between α-MSH-stimulated and non-treated B16-4A5 cells (Table 1). We then focused on the downregulation of miR-141-3p and miR-200a-3p because their nucleotide sequences were identical except for two nucleotides (Fig. 1A) and the target genes of both miRNAs were predicted to be common from TargetScan Mouse 7.1.

**Table 1:** Differentially expressed miRNAs detected by 3D-Gene microarray analysis.

miiRNA sysmatic name	miRBase accession number	Regulation	Ratio	Fold change (Log2)
mmu-miR-10b-3p	MIMAT0004538	up	7.13	2.83
mmu-miR-148a-3p	MIMAT0000516	down	0.13	−2.94
mmu-miR-let-7c-1-3p	MIMAT0004622	down	0.15	−2.68
mmu-miR-218-5p	MIMAT0000663	down	0.17	−2.54
mmu-miR-let-7a-1-3p	MIMAT0004620	down	0.18	−2.47
mmu-miR-31-3p	MIMAT0004634	down	0.20	−2.33
mmu-miR-let-7d-5p	MIMAT0000383	down	0.20	−2.32
mmu-miR-26a-5p	MIMAT0000533	down	0.21	−2.25
mmu-miR-141-3p	MIMAT0000153	down	0.24	−2.05
mmu-miR-16-5p	MIMAT0000527	down	0.25	−2.01
mmu-miR-24-3p	MIMAT0000219	down	0.25	−2.00
mmu-miR-200a-3p	MIMAT0000519	down	0.25	−2.00
mmu-miR-423-5p	MIMAT0004825	down	0.25	−2.00

To confirm the results from the miRNA array analysis, we examined the expression levels of miR-141-3p and miR-200a-3p in α-MSH-stimulated and non-treated B16-4A5 cells by the quantitative real-time polymerase chain reaction (qRT-PCR). As expected, the expression levels of both miRNAs in α-MSH-stimulated cells were significantly downregulated as compared with control non-treated cells. Additionally, we confirmed the downregulation of miR-148a-3p, which targets *Mitf* similar to results reported previously (Fig. 1B and Supplementary Fig. 1).

### Overexpression of miR-141-3p and miR-200a-3p in B16-4A5 cells inhibits α-MSH-stimulated melanogenesis by suppressing *Mitf* expression

**Figure 2:**
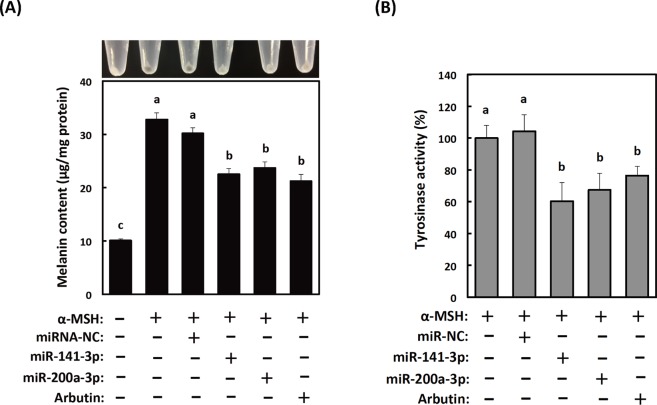
Effects of transfection with mature miR-141-3p and miR-200a-3p on α-melanocyte stimulating hormone (α-MSH)-induced melanogenesis in B16-4A5 cells. (**A**) Colorimetric measurement of the melanin content of B16-4A5 cells from three independent experiments (bottom) and images of one representative assay (top). (**B**) Overexpression of mature miR-141-3p and miR-200a-3p in B16-4A5 cells significantly suppresses tyrosinase activity. The negative control miRNA (miRNA-NC) was designed to have no significant sequence similarity to mouse, rat, or human transcription products. Cells were treated with α-MSH (2 µM) for 72 h. Values of each experiment are expressed as means ± SE of three separate experiments, each performed in triplicate. Means not sharing a common letter within a column are significantly different at p < 0.05. Statistical comparisons were performed using the Tukey-Kramer test.

The melanin content, a marker of melanogenesis, increased with time after α-MSH stimulation. To disclose the role of miR-141-3p or miR-200a-3p in this response, we first measured melanin contents and tyrosinase activities in B16-4A5 cells transfected with miR-141-3p or miR-200a-3p, and with and without α-MSH treatment. As shown in Fig. 2A,B, the melanin contents and tyrosinase activities in transfected cells were suppressed approximately 30%. These were the same inhibition levels achieved following treatment with 200 µM arbutin. In contrast, the melanin content and tyrosinase activity in cells transfected with non-specific control miRNA were not changed compared with untransfected α-MSH-stimulated B16-4A5 cells. Additionally, the melanin contents and tyrosinase activities in cells transfected with non-specific control miRNA, miRNA-141-3p, or miR-200a-3p were also not change compared with untransfected α-MSH-unstimulated B16-4A5 cells (Supplementary Fig. 2). These results suggest that miR-141-3p and miR-200a-3p act as melanogenesis modulators in melanocytes stimulated with α-MSH.

### Expression of the melanogenesis master transcription factor, Mitf, is regulated by miR-141-3p and miR-200a-3p

**Figure 3:**
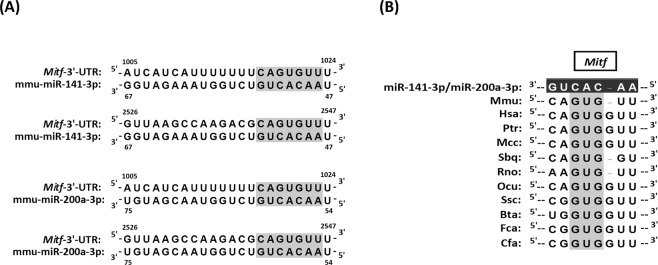
MiR-141-3p and miR-200a-3p target *microphthalmia-associated transcription factor* (*Mitf*) directly in the mouse B16-4A5 melanoma cell line. (**A**) Schematic diagram of putative miR-141-3p and miR-200a-3p binding sites within the mouse *Mitf* 3′-untranslated region (UTR) are shown. Binding site positions are numbered relative to the first nucleotide in each mRNA sequence. The light shadow indicates a seed sequence position in the mouse *Mitf* 3′-UTR. (**B**) Interspecies conservation of putative miR-141-3p and miR-200a-3p binding sites within the *Mitf* 3′-UTR is shown. The dark shadow site denotes perfectly conserved sequences between mouse and other species. Mmu, mouse; Has, human; Ptr, chimpanzee; Mcc, rhesus monkey; Sbq, Bolivian squirrel monkey; Rno, rat; Ocu, rabbit; Ssc, pig; Bta, Cow; Fca, cat; Cfa, dog.

**Figure 4:**
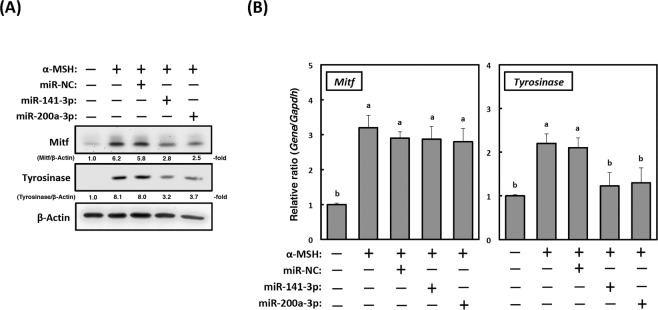
Expression of microphthalmia-associated transcription factor (Mitf) is translationally regulated by miR-141-3p and miR-200a-3p during melanogenesis. (**A**) Mitf and tyrosinase protein expression (20 µg of protein/lane) in B16-4A5 cells transfected with miR-141-3p or miR-200a-3p. Representative western blots from three independent experiments are shown. Cells were treated with α-MSH (2 µM) for 72 h, and then cell lysates were prepared. (**B**) Changes in *Mitf* and *tyrosinase* mRNA expression in cells transfected with miR-141-3p and miR-200a-3p as examined by the quantitative real-time polymerase chain reaction. *Glyceraldehyde-3-phosphate dehydrogenase (Gapdh)* was used as the internal control. Cells were treated with α-MSH (2 µM) for 24 h. Then, total RNA was extracted from the cells by TRIzol reagent. Values for each group are expressed as means ± SE of three independent experiments, each performed in triplicate. Means not sharing a common letter within a column are significantly different at p < 0.05. Statistical comparisons were performed using the Tukey-Kramer test.

To identify potentially relevant target genes of miR-141-3p and miR-200a-3p during α-MSH-stimulated melanogenesis, we searched for candidate genes using the TargetScan Mouse 7.1 database. Both of these miRNAs target about 5,437 genes, and *Mitf* is included as a common target (Fig. 3A). As shown in Fig. 3B, numerous species have the common binding site “GUG” on the 3′-UTR of *Mitf* to miR-141-3p and miR-200a-3p. Therefore, both miRNAs probably act as *Mitf* regulators in cells from numerous species. We next examined the expression levels of Mitf protein and mRNA in α-MSH-stimulated B16-4A5 cells. As shown in Fig. 4A, the levels of Mitf protein were decreased in cells transfected with either miR-141-3p or miR-200a-3p compared with α-MSH-stimulated cells and cells transfected with the non-specific miRNA control. Additionally, the levels of tyrosinase protein were decreased concurrently with Mitf expression. On the other hand, the levels of *Mitf* mRNA in cells transfected with miR-141-3p or miR-200a-3p were unchanged among all cells, but the levels of *Tyrosinase* mRNA were suppressed (Fig. 4B). These results strongly indicate that the downregulation of tyrosinase expression in cells transfected with miR-141-3p or miR-200a-3p is due to transcriptionally controlling Mitf via melanocortin 1 receptor signalling.

### *Mitf* is a common target of miR-141-3p and miR-200a-3p

**Figure 5:**
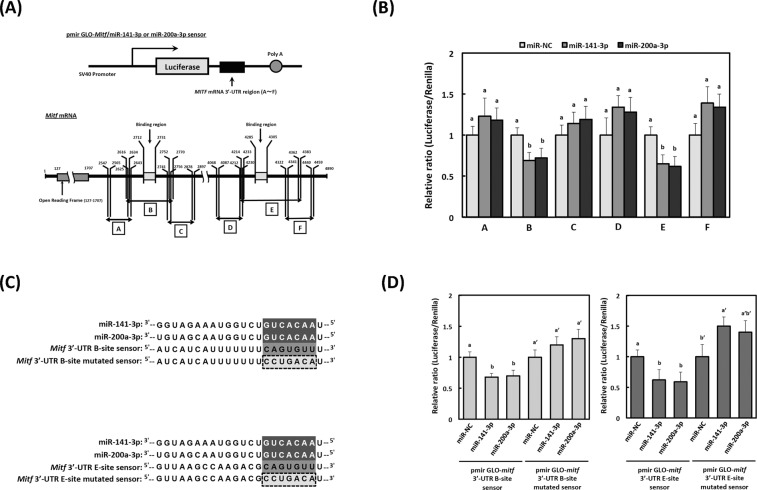
Identification of miR-141-3p and miR-200a-3p target genes by the luciferase reporter assay. (**A**) Schematic representation of the sensor vectors used in the luciferase assay for identification of target regions in *microphthalmia-associated transcription factor (Mitf)* for miR-141-3p and miR-200a-3p. Construction of six different luciferase reporter plasmids (cloning sites A–F) is shown, including the 3′-untraslated region (UTR) of *Mitf* mRNA. (**B**) Luciferase activities were calculated as a ratio of firefly to *Renilla* luciferase activity. Values of each group are expressed as means ± SE of three independent experiments, each performed in triplicate. Means not sharing a common letter within a column are significantly different at p < 0.05. Statistical comparisons were performed using the Tukey-Kramer test. Statistical comparisons were performed using the Tukey-Kramer test. (**C**) The mutated region corresponding to the seed sequences of miR-141-3p and miR-200a-3p sensor vectors were generated from pmir GLO-*Mitf*/miR-141-3p/200a-3p sensor-B or sensor-E. Mutations were introduced into the seed regions of each binding site, as indicated by the dotted box. (**D**) Each sensor vector (pmir GLO-*Mitf*/miR-141-3p/200a-3p sensor-mutated-B or sensor-mutated-E), miR-NC, and miR-141-3p or miR-200a-3p, was co-transfected into MC3T3-E1 cells. Luciferase activities were quantified as the ratio of firefly to *Renilla* luciferase activities. Values for each group are expressed as means ± SE of three independent experiments, each performed in triplicate. Means not sharing a common letter within a column are significantly different at p < 0.05. Statistical comparisons were performed using the Tukey-Kramer test. Statistical comparisons were performed using the Tukey-Kramer test.

To determine the target region of miR-141-3p and miR-200a-3p for *Mitf* mRNA, we constructed six types (A-F) of luciferase reporter plasmids with or without the possible binding regions (site 1: numbers 2712–2731; site 2: numbers 4285–4305) in the 3′-UTR of *Mitf* mRNA (Fig. 5A). The levels of luciferase activity in pmir GLO-*Mitf*/miR-141-3p and miR-200a-3p sensor-B or -E, which contain the 2712–2731 and 4285–4305 regions of the 3′-UTR of *Mitf*, were lower than those in the non-specific control sensor vector (p < 0.05, Fig. 5B). On the other hand, the levels of luciferase activity in pmir GLO-*Mitf*/miR-141–3p and miR-200a-3p sensor-A, -C, -D, or -F, which lack the 2712–2731 and 4285–4305 regions of the 3′-UTR of *Mitf*, were unchanged compared with those in the non-specific control sensor vector. We constructed pmir GLO-*Mitf*/miR-141-3p and miR-200a-3p mutated sensor-B and -E to confirm these observations (Fig. 5C). Luciferase activities in cells transfected with pmir GLO-*Mitf*/miR-141-3p, and miR-200a-3p sensor-B and E (wild-type), were significantly reduced, although in cells transfected with mutants of the seed region the decrease was absent (Fig. 5D). These results strongly suggest that the target region of *Mitf* is the common binding site for miR-141-3p and miR-200a-3p.

### Overexpression of miR-141-3p and miR-200a-3p in reconstructed skin tissues inhibits α-MSH-stimulated melanogenesis

**Figure 6:**
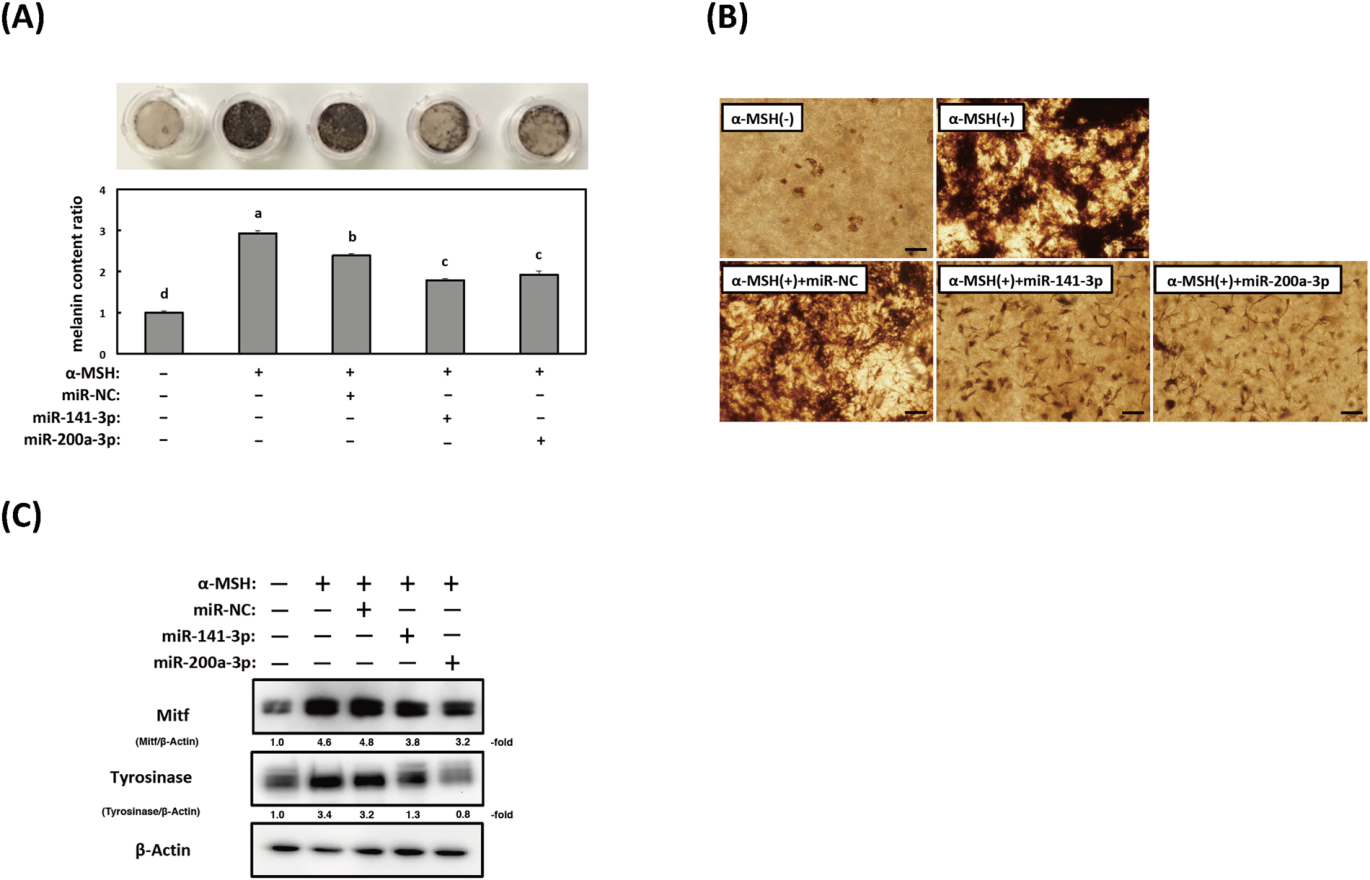
Effects of miR-141-3p and miR-200a-3p on melanin production in reconstructed skin tissue. (**A**) Colorimetric measurement of the melanin content of B16-4A5 cells from three independent experiments (bottom) and images of reconstructed skin tissue of each treatment (top). Values of each group are expressed as means ± SE of three independent experiments, each performed in triplicate. Means not sharing a common letter within a column are significantly different at p < 0.05. Statistical comparisons were performed using the Tukey-Kramer test. Statistical comparisons were performed using the Tukey-Kramer test. (**B**) Microscopic observation of three-dimensional reconstructed skin tissue after treatment without or with miR-141-3p or miR-200a-3p. The skin model was transfected with miR-141-3p or miR-200a-3p after melanogenesis was induced by α-melanocyte stimulating hormone (α-MSH) for 21 days. (**C**) Mitf and tyrosinase protein expression (10 µg of protein/lane) in a three-dimensional tissue culture model of human epidermis transfected with miR-141-3p or miR-200a-3p. Representative western blots from three independent experiments are shown.

We assessed whether miR-141-3p and miR-200a-3p were involved in α-MSH-stimulated melanogenesis by using a three-dimensional tissue culture model of human epidermis (3D-MHE). After culturing for 21 days with EPI-100LLMM medium, skin tissues transfected with miR-141-3p and miR-200a-3p and stimulated with α-MSH exhibited markedly decreased melanin content (by approximately 25–35%) when compared with α-MSH-stimulated 3D-MHE or 3D-MHE transfected with non-specific control miRNA (Fig. 6A,B). The expression levels of both the Mitf and tyrosinase proteins were also decreased in 3D-MHE transfected with either miR-141-3p or miR-200a-3p compared with α-MSH-stimulated 3D-MHE and 3D-MHE transfected with the non-specific miRNA control (Fig. 6C). The changes in protein expression of Mitf and tyrosinase using a 3D-MHE model showed the same tendency as the results using B16-4A5 cells.

## Discussion

**Figure 7:**
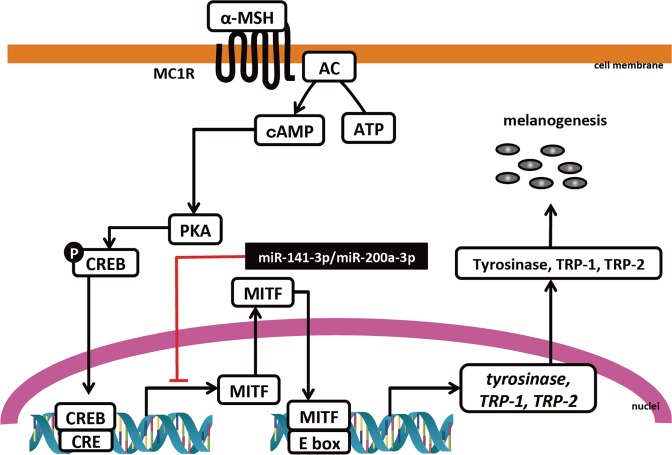
Schematic overview of the regulatory mechanisms of miR-141-3p and miR-200a-3p in α-melanocyte stimulating hormone (α-MSH)-stimulated melanogenesis. (The double helix drawing in Fig. 7 has been obtained from Database Center for Life Science, http://togotv.dbcls.jp/ja/togopic.2018.23.html. DCLB is licensed under Creative Commons Attribution 4.0 International license (CC-BY-4.0). TOGO picture gallery in DCLB is released free of charge so that anyone can view and use it freely).

The purpose of this study was to show that miR-141-3p and miR-200a-3p regulate α-MSH-stimulated melanogenesis. We demonstrated that overexpression of both miR-141-3p and miR-200a-3p significantly decreased melanin content and tyrosinase activity. Furthermore, we also identified *Mitf* as a common target for miR-141-3p and miR-200a-3p during α-MSH-stimulated melanogenesis by using a luciferase reporter assay. The binding of miR-141-3p and miR-200a-3p to the *Mitf* 3′-UTR was prevented by including a point mutation in each individual binding region of the *Mitf* 3′-UTR. These results suggest that changes of endogenous miR-141-3p and miR-200a-3p levels are closely related to α-MSH-stimulated melanogenesis (Fig. 7).

MITF plays a central role in melanogenesis signalling in melanocytes. It is a member of the MYC family of basic helix-loop-helix leucine zipper transcription factors and is most closely related to transcription factor E3, transcription factor EC, and transcription factor proteins^33^. MITF expression is regulated by at least four different transcriptional molecules (paired box family of transcription factor 3, sex determining region Y-box 10, Wnt/β-catenin pathway effector lymphoid enhancer-binding factor 1, and cAMP pathway effector cAMP response element binding protein)^34^. Nine isoforms have been reported in humans, each with a different 5′ specificity (MITF-A, -B, -C, -D, -E, -H, -J, -M, and -MC)^35^. Tachibana *et al*., and other researchers, demonstrated that melanocyte-specific MITF (MITF-M) consists of 419 amino acid residues and its mRNA expression is detected exclusively in melanocytes and pigmented melanoma cells^36,37^. MITF-M transactivates several genes involved in melanogenesis, such as melanogenic enzymes (tyrosinase, TRP-1 and DCT or TRP-2, respectively), melanosome biogenesis-related proteins (pre-melanosome protein 17, G protein-coupled receptor 143, solute carrier family 24 member 5, and melanosome transport proteins, which are members of the RAS oncogene family (RAB7A, RAB27A)), melanosome delivery proteins (adaptor-related protein complex 1 subunits (AP1B1, AP1G1, AP1S1, AP1S2)), subunit 1 BLOC3 complex (BLOC3S1), subunit 1 BLOC2 complex (BLOC2S1), BLOC3 complex subunits 1, 2, and 3, and membrane proteins (melanocortin 1 receptor, endothelin receptor type B, c-Kit receptor, and melastatin)^38^. From these findings, MITF has become a potential therapeutic target for skin pigmentation disorders or whitening.

The miRNA-200 family is highly conserved among vertebrate species and consists of five members that form two clusters located in two different chromosomes^39^. Cluster I miRNA-200s (miR-200b/-200a/-429) are located in an intergenic region of chromosome 1 in humans and on chromosome 4 in mice. Cluster II miRNA-200s (miR-200b/-200a/-429) are located on chromosome 12 in humans and chromosome 6 in mice^40^. Furthermore, the miRNA-200 family can be divided into two functional groups based on their similar seed sequences. One is composed of miR-200b, miR-200c, and miR-429, and the other is composed of miR-141 and miR-200a, each having a similar seed sequence^41^. The difference between the seed sequences in both groups is only one nucleotide. Therefore, it can be predicted that the miRNA-200 family targets many common genes to increase the efficiency of genetic regulation.

It is widely understood that miRNA-200 family members serve as crucial molecules for tumorigenesis, cell growth, angiogenesis, and the metastasis processes in each type of cancer cell^42–45^. In addition, several developmental studies show that the miRNA-200 family forms integral components of various biological processes including inflammation^46^, adipocyte differentiation^47^, placental development^48^, aging^49^, bone metabolism^50,51^, and calcification^52^. Our team has shown, for the first time, that miR-141-3p and miR-200a-3p regulate pre-osteoblast differentiation through the direct targeting of Dlx5^53^. There is, however, very little information at present regarding the relationship between miR-141-3p/miR-200a-3p and skin disorders.

A decrease of UV-B-induced extracellular signal-regulated kinase/Akt-dependent phosphatase and tensin homolog deleted on chromosome 10 (PTEN) levels promotes survival of epidermal keratinocytes^54^. PTEN also negatively regulates UV-B-induced DNA damage repair in human HaCaT keratinocytes by limiting xeroderma pigmentosum C expression^55^. Li *et al*. showed that downregulation of PTEN expression by UV-B irradiation was controlled by up-regulating miR-141 in HaCaT cells^56^. The results of the current study reveal the mechanisms by which miR-141-3p and miR-200a-3p transcriptionally regulate melanogenesis; i.e. direct targeting of *Mitf*. This adds miR-141-3p and miR-200a-3p to the previously reported group of miRNAs that control melanogenesis. From these reports, it is conceivable that controlling miR-141-3p and/or miR-200-3p is a promising approach for treating photodamage.

Although MITF small interfering RNA creams have been used safely for melasma in the clinical setting, the effects of these agents are extremely low owing to the difficulty of RNAi-materials penetrating through the stratum corneum barrier of the skin^57^. Hence, several studies are ongoing to develop revolutionary RNAi-based therapeutics that eliminate the drawbacks of conventional methods. For example, ultra-deformable, elastic, and surfactant-ethanol-cholesterol liposomes improve transfection and delivery efficiencies^18^. Additionally, conjugates of cell-penetrating peptides or chitosan with miRNA also improve transfection and delivery efficiencies^18^. According to the ZION market research report, the global skin lightening products market was calculated to be around 4 billion United States dollars in 2017 and was predicted to reach approximately 8.9 billion dollars by 2024. Thus, the pharmaceutical and cosmetic industries will continue to seek to develop optimally effective RNAi-based therapeutics for topical application.

## Conclusion

Our findings reveal that miR-141-3p and miR-200a-3p regulate skin pigmentation by controlling Mitf expression in mouse melanocytes and a three-dimensional tissue culture model of human epidermis. Therefore, miRNAs may be beneficial molecules for whitening.

## Materials and Methods

### Materials

α-MSH was purchased from Sigma-Aldrich (St. Louis, MO, USA). Low glucose Dulbecco’s modified Eagle’s medium (DMEM-LG), OPTI-MEM, foetal bovine serum (FBS), and Lipofectamine™ RNAi MAX were purchased from Invitrogen (Carlsbad, CA, USA). The antibody to mouse Mitf was obtained from EMD Millipore (Billerica, MA, USA). The antibody to mouse tyrosinase was obtained from Abcam (Cambridge, UK). The antibody to mouse β-actin was obtained from Cell Signaling Technology (Danvers, MA, USA).

### Cell culture and α-MSH stimulation

Mouse B16 melanoma cells (4A5) were obtained from the RIKEN Cell Bank (Tsukuba, Ibaraki, Japan). Cells were cultured in a humidified atmosphere at 37 °C with 5% CO_2_ in DMEM-LG supplemented with 10% heat-inactivated FBS, 50 U/mL penicillin, and 100 µg/mL streptomycin. All melanogenesis experiments were performed using B16-4A5 cells within 15 passages. For each experiments, cells were stimulated with 2 µM of α-MSH.

### MiRNA array hybridisation

MiRNA profiling following α-MSH stimulation was carried out with 3D-Gene^®^ Mouse miRNA Oligo chips, ver. 21 (Toray Industries, Kamakura, Japan), that detect 1,900 miRNA transcripts. After α-MSH stimulation for 24 h, total RNA was extracted from B16-4A5 cells using the mirVana miRNA Isolation Kit (Applied Biosystems, Foster City, CA, USA) according to the manufacturer’s protocol. Extracted total RNA was checked with a Bioanalyzer (Agilent Technologies, Santa Clara, CA, USA). Microarray analysis was performed with a 3D-Gene Mouse Oligo chip 24 k (Toray Industries). For efficient hybridization, this microarray adopts a columnar structure to stabilize spot morphology and enable micro-beads agitation. Total RNA was labelled with Cy5 using the Amino Allyl MessageAMP II aRNA Amplification Kit (Applied Biosystems). The Cy5-labeled a RNA pools were mixed with hybridization buffer, and hybridized for 16 h. Hybridization was performed according to the supplier’s protocols (www.3d-gene.com). Hybridization signals were obtained by using a 3D-Gene Scanner, and processed by 3D-Gene Extraction software (Toray Industries). Detected signals for each gene were normalized by the global normalization method; the median of the detected signal intensity was adjusted to 25.

Biological replicates were not prepared for microarray analysis. Technical and biological replicates were prepared for the qRT-PCR validation experiments.

### QRT-PCR

To confirm reproducibility of the miRNA expression profiles obtained by the miRNA array analysis, we used TaqMan^®^ miRNA reverse transcription and TaqMan^®^ miRNA assay kits (Applied Biosystems). After α-MSH stimulation for 24 h, total RNA was extracted from the cells by TRIzol reagent containing phenol/guanidine isothiocyanate (Invitrogen) with DNase I treatment. Complementary DNA (cDNA) was then synthesized using reverse transcriptase with 12.5 ng of total RNA. The products were subjected to qRT-PCR using an Applied Biosystems StepOne™ RT-PCR system. Expression levels were normalized to U6 as the internal control and quantified by the comparative Ct (ΔΔCt) method. QRT-PCR consisted of 45 cycles of 95 °C for 10 sec, 60 °C for 40 sec, and 72 °C for 1 sec, after an initial denaturation step (95 °C for 10 min).

To determine the levels of *Mitf* and *Tyrosinase* mRNAs, we prepared cDNA from total RNA samples using a high capacity RNA-to-cDNA kit (Applied Biosystems). Subsequently, qRT-PCR was performed using the SYBR™ Green PCR Master Mix Kit. Primers for *Mitf, tyrosinase*, and *glyceraldehyde-3-phosphate dehydrogenase* (*Gapdh*) were purchased from Takara Bio (Kusatsu, Japan, primer sequences are shown in Supplementary Table 1). The expression level of each gene was determined using the ΔΔCt method and normalized to that of *Gapdh*, which was used as the internal control. The PCR reaction consisted of 45 cycles of 95 °C for 15 s and 60 °C for 60 s, after an initial denaturation step (95 °C for 10 min).

### Transfection of B16-4A5 cells with miRNAs

B16-4A5 cells were seeded into six-well plates at 1 × 10^5^ cells/mL/well the day before transfection. Mature miR-141-3p, miR-148-3p, and miR-200a -3p (40 nM, Applied Biosystems; Fig. 1A) were transfected into the cells using cationic liposomes (RNAiMAX) according to the manufacturer’s lipofection protocol (Thermo Fisher Scientific, Waltham, MA, USA). The transfection efficiency was >80% as evaluated by transfecting cells with an Alexa 488-conjugated dextran probe. Non-specific miRNA (Applied Biosystems) was used as the control for non-specific effects.

### Determination of melanin content

After α-MSH stimulation for 72 h, B16-4A5 cells were harvested with the culture medium using a rubber tipped cell scraper. The cell pellet was collected by centrifugation (5,000 × g for 15 min at 4 °C). The supernatants was transferred to an ultracentrifugation tube and centrifuged at 100,000 × g for 30 min at 4 °C for purification of secreted melanosomes (OptimaTM TLX ultracentifuge (Rotor: TLA-110), Beckman Coulter, CA, USA). The cell pellet containing crude melanosomes was lysed in 1 N NaOH for 1 h at 60 °C. The melanin content was measured at 475 nm using a Varioskan-LUX multimode microplate reader (Thermo Fisher Scientific) and was calculated from a standard curve of synthetic melanin.

### Measurement of tyrosinase activity

After α-MSH stimulation for 72 h, B16-4A5 cells were lysed in ice-cold lysis buffer (10 mM Tris-HCl (pH 7.5), 1% NP-40, 0.1% sodium deoxycholate, 0.1% sodium dodecyl sulphate (SDS), 150 mM NaCl, 1 mM EDTA) containing a complete protease inhibitor mixture. Cell lysates were centrifuged at 15,000 × g for 30 min at 4 °C and the supernatant used as a crude tyrosinase solution. The crude tyrosinase solution containing 0.05% L-dopa was mixed with 50 mM potassium phosphate buffer (pH 6.8), then reacted for 30 min at 37 °C. After incubation, dopachrome formation was measured at 475 nm using a Varioskan-LUX multimode microplate reader.

### Western blotting

For preparation of cell lysates, B16-4A5 cells, stimulated by α-MSH for 72 h were washed twice with PBS, then harvested with a cell scraper. The cell pellet after centrifugation at 15,000 × g for 30 min at 4 °C was resuspended in radioimmunoprecipitation assay buffer containing protease (25 × Complete^®^) and phosphatase inhibitors (Sigma-Aldrich). The protein content was measured with a DC protein assay kit (Bio-Rad, Hercules, CA, USA). Each whole cell lysate was resuspended in SDS-polyacrylamide gel electrophoresis (PAGE) buffer containing 2% mercaptoethanol and boiled at 98 °C for 5 min. Protein samples were subjected to SDS-PAGE in a 12% polyacrylamide gel and subsequently electroblotted onto a polyvinylidene difluoride membrane (GE Healthcare, Pittsburgh, PA, USA). After blocking non-specific binding sites for 1 h with 5% non-fat milk in TBST (tris-buffered saline containing 0.1% Tween 20), the membrane was incubated overnight at 4 °C with the various primary antibodies. The membrane was then washed 3 times in TBST, incubated further with a horseradish peroxidase-conjugated secondary antibody at room temperature, then washed again 3 times in TBST. Protein bands were detected using an enhanced chemiluminescence kit (GE Healthcare) and chemiluminescence detector (Davinchi Chem, Fujifilm Wako Pure Chemical, Osaka, Japan).

### Luciferase assay

To determine targets for miR-141-3p and miR-200a-3p in *Mitf*, we constructed six different pmir GLO-*Mitf*/miR-141-3p and miR-200a-3p sensor plasmids by inserting candidate binding sites in the 3′-UTR of *Mitf* mRNA into the Dra I and Xba I sites of the pmir. The PCR primer sequences used for construction of the sensor plasmids were: pmir GLO-*Mitf*/miR-141-3p/200a-3p sensor-A sense, 5′-AGCTTTGTTTAAACGGCGCGCCGGTGAGGACACCGGATGTTTG-3′; antisense, 5′-GCTCTAGAGCAGAAATGTGCCCAGTTCAG-3′; pmir GLO-*Mitf*/miR-141-3p/200a-3p sensor-B sense, 5′-AGCTTTGTTTAAACGGCGCGCCGGTAAAGACAAACTGAACTGGG-3′; antisense, 5′-GCTCTAGAGCACTCACTGTTTTAGATGC-3′; pmir GLO-*Mitf*/miR-141-3p/200a-3p-C sense, 5′-AGCTTTGTTTAAACGGCGCGCCGGTGTAAGAATATGCATC-3′; antisense, 5′-GCTCTAGAGCCAAGAGATAGTGGAATATAC-3′; pmir GLO-*Mitf*/miR-141-3p/200a-3p sensor-D, 5′-AGCTTTGTTTAAACGGCGCGCCGGCAACTTTCTAGGAAGCTTTG-3′; antisense, 5′-GCTCTAGAGCTTCCCTATGTTGTCACCTC-3′; pmir GLO-*Mitf*/miR-141-3p/200a-3p sensor-E sense, 5′-AGCTTTGTTTAAACGGCGCGCCGGGGTGACAACATAGGGAA-3′; antisense, 5′-GCTCTAGAGCGAGATACAATTCTGCACCCAGG-3′; pmir GLO-*Mitf*/miR-141-3p/200a-3p sensor-F sense, 5′-AGCTTTGTTTAAACGGCGCGCCGGGGTGACAACATAGGGAA-3′; antisense, 5′-GCTCTAGAGCGAGATACAATTCTGCACCCAGG-3′.

We also constructed vectors with mutated miR-141-3p/200a-3p complementary sites in *Mitf* by using a PrimeStar^TM^ mutagenesis basal kit (Takara Bio). The PCR primer sequences used for construction of the mutated pmir GLO-*Mitf*/miR-141-3p/200a-3p sensor-B and sensor-E plasmid were: pmir GLO-*Mitf*/miR-141-3p/200a-3p sensor-mutated-B sense, 5′-TTTTTTCCTGACATGTATTAATTTGTAAGAA-3′; antisense, 5′-TAATACATGTCAGGAAAAAAATGATGATAAT-3′; pmir GLO-*Mitf*/miR-141-3p/200a-3p sensor-mutated-E sense, 5′-AAGACGCCTGACATTTGCTCACTGTTTTTAA-3′; antisense, 5′-GAGCAAATGTCAGGCGTCTTAACTTAACCAG-3′.

B16-4A5 cells were inoculated into 12-well plates (1 × 10^5^ cells/mL), transfected with the sensor vector or mutated sensor vector plasmid and 40 nM of miR-141-3p or miR-200a-3p using Lipofectamine^TM^ RNAiMAX, then cultured for 24 h. Luciferase activity was measured using a luciferase assay system according to the manufacturer’s protocol (Promega, Madison, WI, USA). Relative luciferase activity is expressed as the ratio of measured luciferase activity to the control (non-specific miRNA).

### Reconstructed skin model

3D-MHE from a black donor (MEL-300-B, lot no. 28492) was purchased from Kurabo Industries (Osaka, Japan). Briefly, human epidermal keratinocytes and normal human melanocytes derived from dark skin donors were co-cultured on a collagen-coated membrane. After delivery from Kurabo, the *in vitro* skin model was immediately precultured for 12 h with DMEM/high glucose medium supplemented with 10% FBS, after which the tissue culture was washed twice with PBS, and transferred to a 12-well microplate.

Mature type of miR-141-3p and miR-200a-3p (40 nM, Applied Biosystems), that were designed to bind to and inhibit the activity of endogenous specific miRNAs when introduced into cells, were transfected from both sides (apical side: 12 h; basolateral side: 36 h: 12 h × 3, exchanging transfection medium every 12 h) by using cationic liposomes (RNAiMAX) according to the manufacturer’s lipofection protocol. Non-specific control miRNA was used for detecting non-specific effects. Forty-eight hours after transfection, the tissue was cultured in EPI-100LLMM medium (MatTek, Ashland, MA, USA) at 37 °C with 5% CO_2_. Tissue cultures were maintained for up to 21 days. At the end of the culture period, tissues were washed twice with PBS, then fixed with 10% formalin. The collagen-coated membrane was removed from the culture chamber and observed under a fluorescence microscope (FX-100, Olympus, Tokyo, Japan). After microscopic observation, the tissue was divided in half with a scalpel. One portion was subjected to melanin quantification and the other to western blotting. To measure the melanin content, tissues were lysed in 1 N NaOH for 1 h at 60 °C. The melanin content was measured by the same method as used for cultured cells.

Protein was extracted from tissue samples using the Minute™ Protein Extraction Kit (Invent Biotechnologies, Plymouth, MN, USA), and used for western blotting of Mitf and tyrosinase proteins.

### Statistical analyses

All data were analysed using a statistical analysis software package for Macintosh, version 2.0 (Esumi, Tokyo, Japan). Data are expressed as means ± standard error. Statistical comparisons among multiple groups were performed using the Tukey–Kramer test or between two groups using Student’s *t*-test. Differences were considered significant when *p*-values were less than 0.05.

## Supplementary information

Supplementary information.
