# Supplementary material for: MicroRNA-141-3p and microRNA-200a-3p regulate α-melanocyte stimulating hormone-stimulated melanogenesis by directly targeting microphthalmia-associated transcription factor
Source: Sci Rep. 2020 Feb 7;10:2149. doi: 10.1038/s41598-020-58911-w (PMC7005774; doi:10.1038/s41598-020-58911-w)
Supplement: Supplementary file 1 — Supplementary information. [file 41598_2020_58911_MOESM1_ESM.pdf]

Supplementary information

MicroRNA-141-3p and microRNA-200a-3p regulate α-melanocyte stimulating hormone-stimulated melanogenesis by directly targeting microphthalmia-associated transcription factor

Tomohiro Itoh[1]*, Kanako Fukatani[2], Ayaka Nakashima[3], Kengo Suzuki[3]

[1] Laboratory for Molecular Chemistry of Aquatic Materials, Department of Life Sciences, Graduate School of Bioresources, Mie University, 1577 Kurimamachiya, Tsu, Mie 514-8507, Japan
[2] Laboratory for Molecular Chemistry of Aquatic Materials, Faculty of Bioresources, Mie University, 1577 Kurimamachiya, Tsu, Mie 514-8507, Japan
[3] Euglena Co., Ltd., Central Research Centre, 75-1 Ono, Tsurumi-ku, Yokohama, Kanagawa 230-0046, Japan
**Correspondence should be addressed to*: Tomohiro Itoh
1577 Kurimamachiya, Tsu, Mie 514-8507, Japan
Tel: 81-59-231-9675; Fax: 81-59-4231-9675
E-mail: titoh@bio.mie-u.ac.jp

supplementaly data figure and Raw data figures

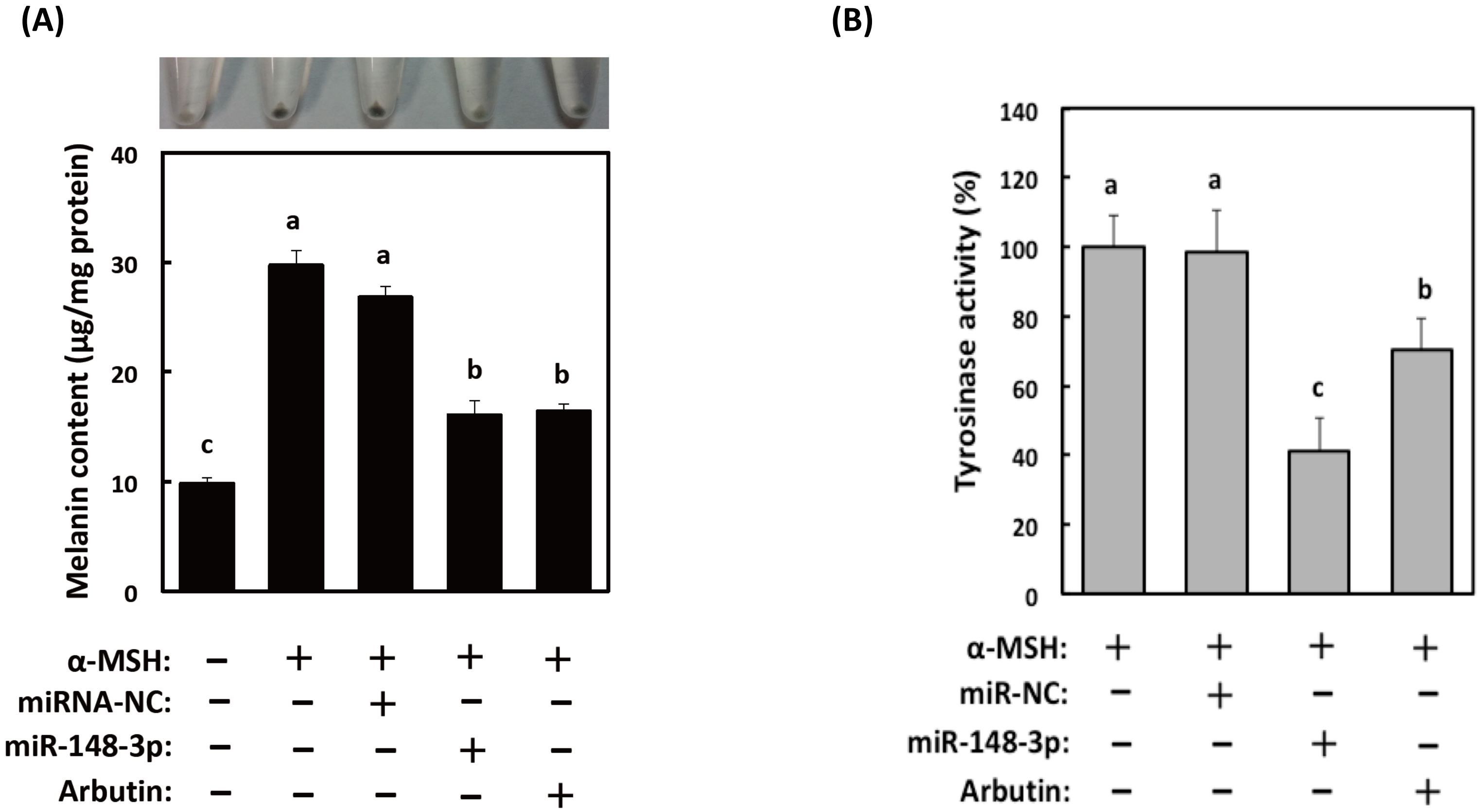

Supplementary Figure 1: Effects of transfection with mature miR-148a-3p on α-melanocyte stimulating hormone (α-MSH)-induced melanogenesis in B16-4A5 cells. (A) Colorimetric measurement of melanin content of B16-4A5 cells from three independent experiments (bottom) and images of one assay (top). (B) Overexpression of mature miR-148-3p in B16-4A5 cells significantly suppresses α-MSH-induced tyrosinase activity. fNegative control miRNA (miRNA-NC) was designed to have no significant sequence similarity to mouse, rat, or human transcription products. Values of each experiment are expressed as means ± SE of three separate experiments, each performed in triplicate. Means not sharing a common letter within a column are significantly different at $p<0.05$. Statistical comparisons were performed using the Tukey-Kramer test.

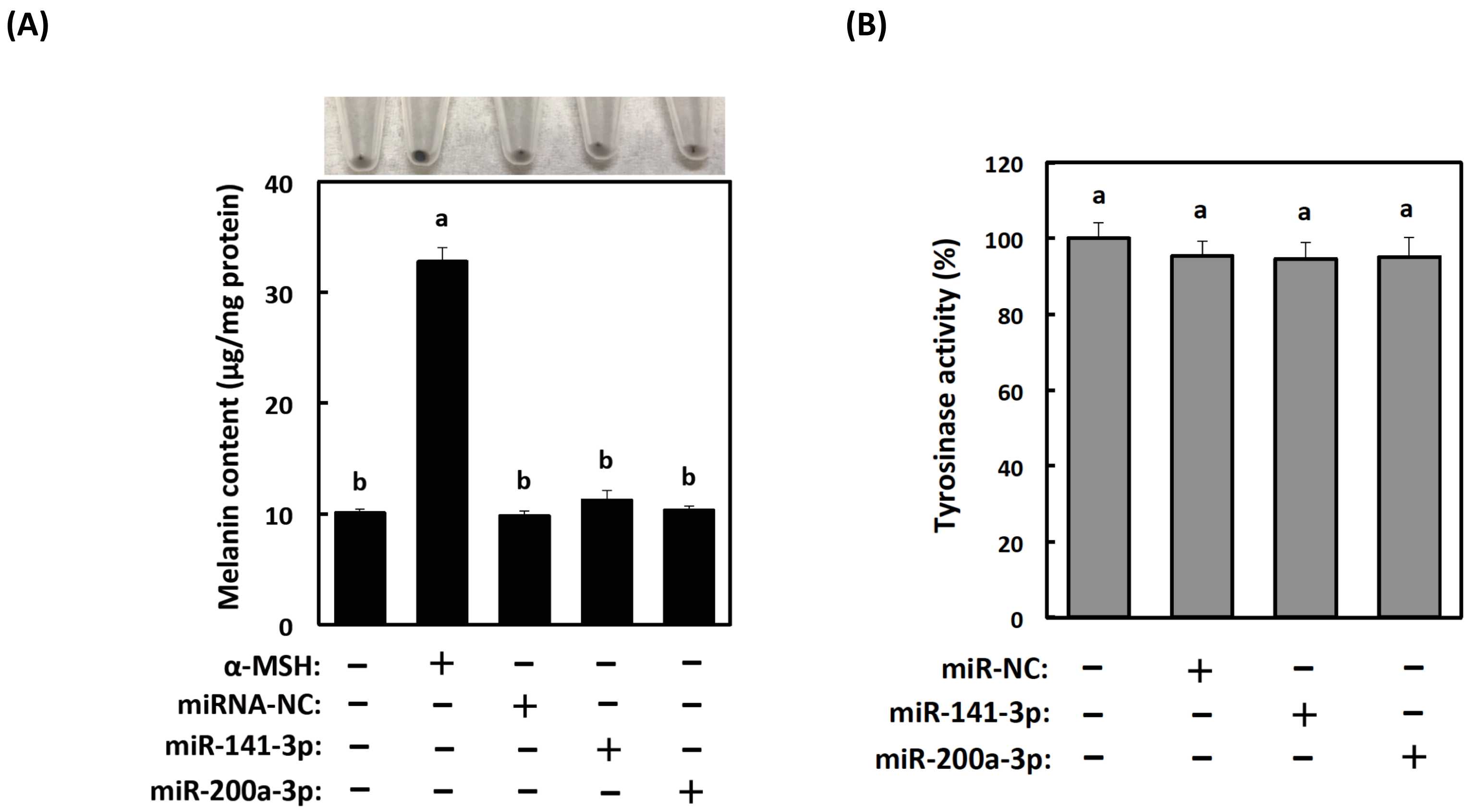

Supplementary Figure 2: Melanin contents were not different among cells transfected with mature miR-141-3p, miR-200a-3p and miR-Negative control. (A) Colorimetric measurement of the melanin content of B16-4A5 cells from three independent experiments (bottom) and images of one assay (top) are shown. (B) Tyrosinase enzyme activity in cells transfected with mimic miRNA is not affected in the absence of stimulation with α-MSH. Negative control miRNA (miRNA-NC) was designed to have no significant sequence similarity to mouse, rat, or human transcription products. Values of each experiment are expressed as means ± SE of three independent experiments, each performed in triplicate. Means not sharing a common letter within a column are significantly different at $p<0.05$. Statistical comparisons were performed using the Tukey-Kramer test.

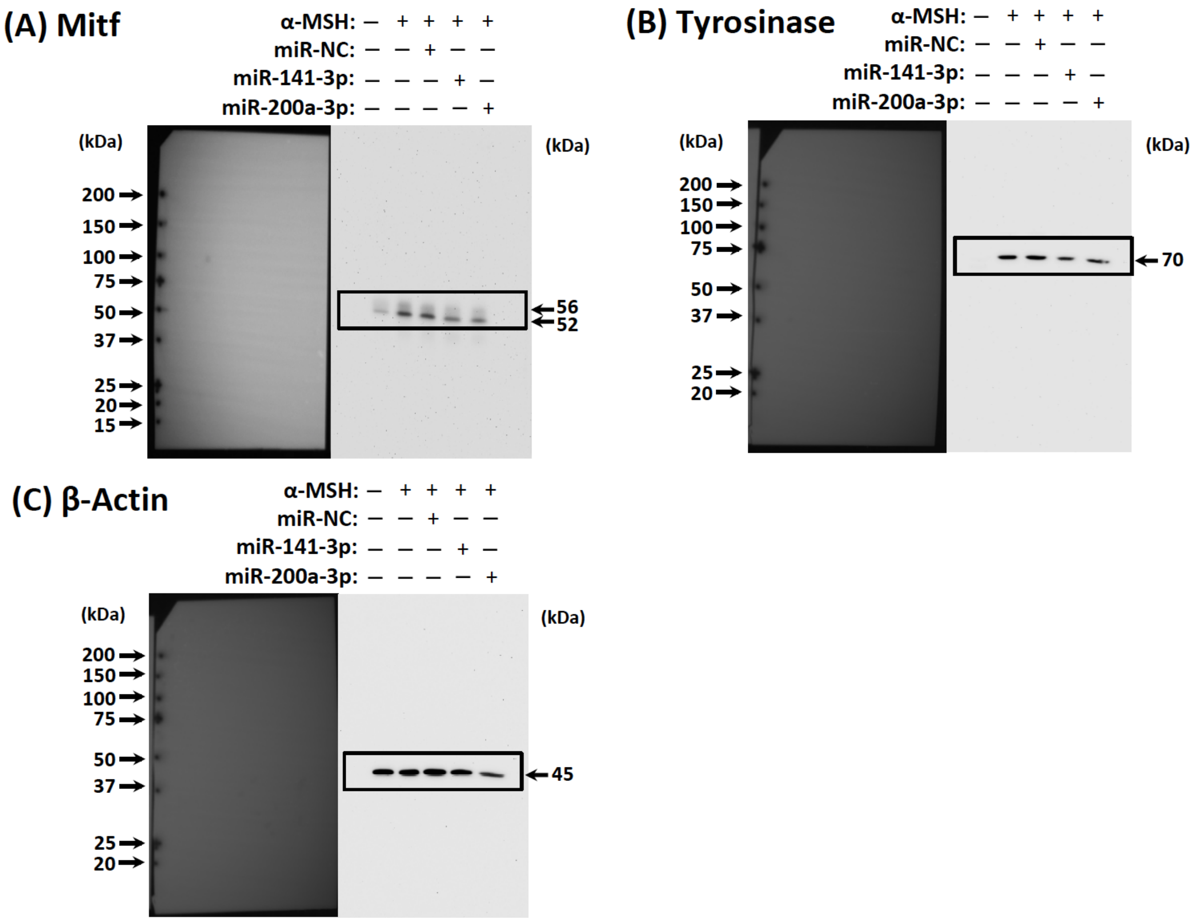

Supplementary Figure 3: Full-length western blot data presented in Figure 4A.

Raw data of full-length western-blots of Mitf (A, primary antibody: anti-microphthalmia (Mi), clone 5, Millipore Cat. No. MAB3747), tyrosinase (B, primary antibody: anti-tyrosinase, Abcam Cat. No. ab52493), and β-actin (C, primary antibody: anti-β-actin, Cell Signaling Technology Cat. No. 4967). (shown as cropped images in Figure 4A).

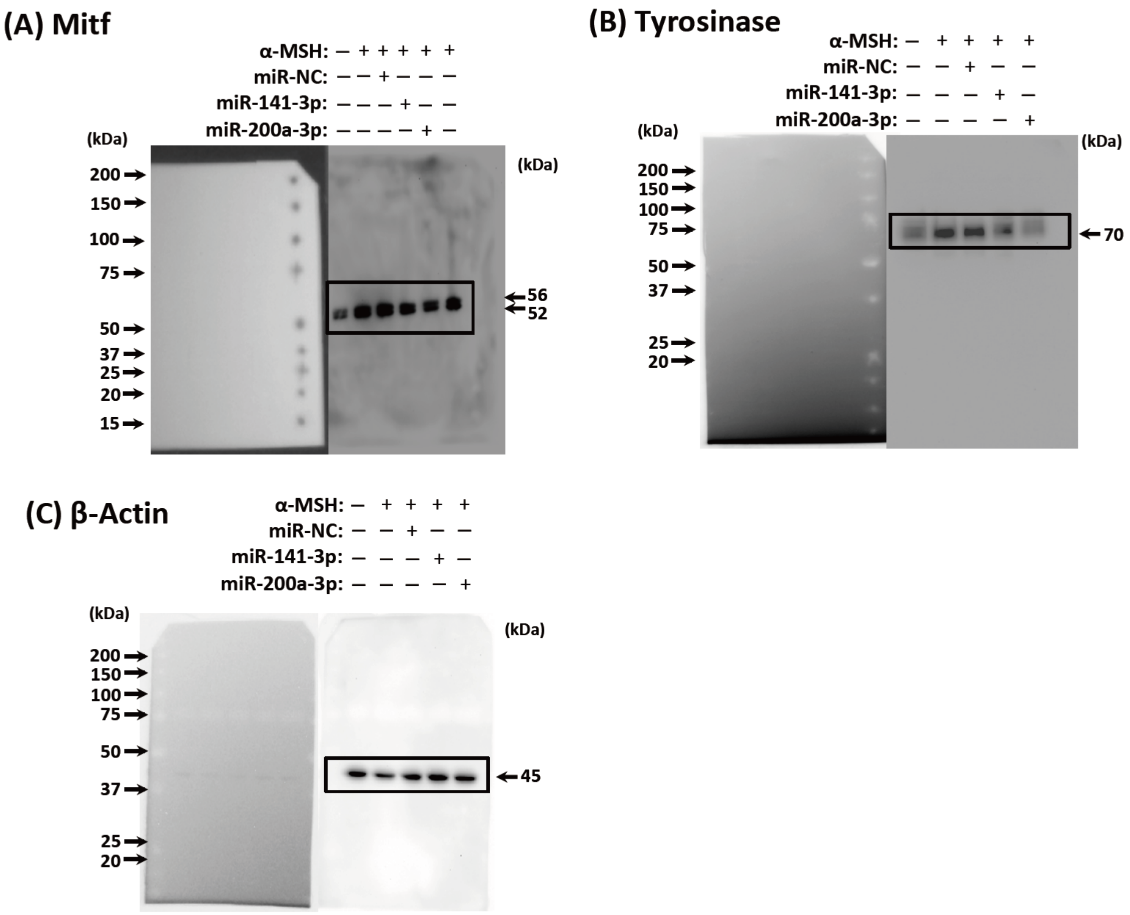

Supplementary Figure 4: Full-length western blot data presented in Figure 6C.
Raw data of full-length western-blots of Mitf (A, primary antibody: anti-microphthalmia (Mi), clone 5, Millipore Cat. No. MAB3747), tyrosinase (B, primary antibody: anti-tyrosinase, Abcam Cat. No. ab52493), and β-actin (C, primary antibody: anti-β-actin, Cell Signaling Technology Cat. No. 4967). (shown as cropped images in Figure 6C).

Supplementary Table 1: List of prime sequences used for quantitative real time PCR analysis in this study.

Genes	Primer sequences (5′ → 3′)
Mitf	5′ - A C T A T G G C C A A G G C A G A G C A A C - 3′
	5′ - T C A G A G G C T A C A A G C C A A G G T A A T G - 3′
Tyrosinase	5′ - C A A G T A C A G G G A T C G G C C A A C - 3′
	5′ - G G T G C A T T G G C T T C T G G G T A A - 3′
Gapdh	5′ - A A A T G G T G A A G G T C G G T G T G A A C - 3′
	5′ - C A A C A A T C T C C A C T T T G C C A C T G - 3′

F: Forward primer, R: Reverse primer
